# Error in Methods

**DOI:** 10.1001/jamanetworkopen.2026.29695

**Published:** 2026-07-29

In the Original Investigation titled “Limbic System Microstructure in Neonates With Antenatal Opioid Exposure,”^1^ published July 2, 2026, the statement of the derivation of adjusted diffusion metrics in Methods should have listed all of these metrics. The article has been corrected.^1^
